# Seasonal changes in the antibody responses against *Plasmodium falciparum* merozoite surface antigens in areas of differing malaria endemicity in Indonesia

**DOI:** 10.1186/1475-2875-12-444

**Published:** 2013-12-09

**Authors:** Supargiyono Supargiyono, Michael T Bretscher, Mahardika A Wijayanti, Inge Sutanto, Dian Nugraheni, Royhan Rozqie, Ayleen A Kosasih, Sulistyawati Sulistyawati, William A Hawley, Neil F Lobo, Jackie Cook, Chris J Drakeley

**Affiliations:** 1 Center for Tropical Medicine, Faculty of Medicine, Gadjah Mada University, Jln Teknika Utara, Barek, Yogyakarta 55281, Indonesia; 2 MRC Centre for Outbreak Analysis and Modelling, Department of Infectious Disease Epidemiology, Imperial College London, St Mary’s Campus, Norfolk Place, London W2 1PG, UK; 3 Department of Parasitology Faculty of Medicine, University of Indonesia, Jln Salemba Raya, Jakarta, Indonesia; 4 Department of Public Health Science, Faulty of Public Health, Ahmad Dahlan University, Jln Prof Dr Soepomo, Warungboto, Umbulharjo, Yogyakarta, Indonesia; 5 United Nations Children Fund (UNICEF), Jakarta, Indonesia; 6 Eck Institute for Global Health, University of Notre Dame, Notre Dame, IN 46556, USA; 7 Malaria Research Unit, Department of Medicine Solna, Karolinska Institutet, Stockholm, Sweden; 8 Department of Immunology & Infection, London School of Hygiene and Tropical Medicine, London W1CE 7HT, UK

**Keywords:** Antibody titre, Seroprevalence, Sero-epidemiology, Malaria transmission, PfMSP1-19, PfAMA1

## Abstract

**Background:**

The transmission of malaria in Indonesia is highly heterogeneous spatially and seasonally. Anti-malaria antibody responses can help characterize this variation. In the present study antibody responses to *Plasmodium falciparum* MSP-1 and AMA-1 were measured to assess the transmission intensity in a hypo-endemic area of Purworejo and a meso-endemic area of Lampung during low and high transmission seasons.

**Methods:**

Filter-paper blood spot samples collected from Purworejo and Lampung by cross-sectional survey during high and low transmission season were stored at −20°C. Indirect ELISA assays were carried out using PfMSP1-19 and PfAMA1 antigens. A positivity threshold was determined by samples from local unexposed individuals, and the differences in seroprevalence, antibody level and correlation between antibody level and age in each site were statistically analysed.

**Results:**

Prevalence of antibodies to either PfMSP1-19 or PfAMA1 was higher in Lampung than in Purworejo in both the low (51.3 *vs* 25.0%) and high transmission season (53.9 *vs* 37.5%). The magnitude of antibody responses was associated with increasing age in both sites and was higher in Lampung. Age-adjusted seroconversion rates showed an approximately ten-fold difference between Lampung and Purowejo. Two different seroconversion rates were estimated for Lampung suggesting behaviour-related differences in exposure. In both settings antibody responses to PfMSP1-19 were significantly lower in the low season compared to the high season.

**Conclusion:**

Seasonal changes may be detectable by changes in antibody responses. This is particularly apparent in lower transmission settings and with less immunogenic antigens (in this case PfMSP1-19). Examination of antibody levels rather than seroprevalence is likely to be a more sensitive indicator of changes in transmission. These data suggest that sero-epidemiological analysis may have a role in assessing short-term changes in exposure especially in low or seasonal transmission settings.

## Background

Malaria still constitutes a major health problem in Indonesia. With regard to the incidence, the country can be divided into two regions, one being the Java and Bali islands and the other being the outer islands. Malaria is hypo-endemic in Java and Bali, which represent only 7% of the land area in Indonesia but are inhabited by approximately 60.0% of the total population. The outer Islands are sparsely populated and the malaria situation varies from hypo to hyper-endemic. With continuous control programmes the national prevalence of malaria decreased from 1.39% in 2007 to 0.6% in 2010 [1]. However, a number of provinces in all regions still have malaria prevalence above the national level. The provinces with the highest prevalences are West Papua (10.6%), Papua (10.1%) and East Nusa Tenggara (4.4%) [1,2].

*Plasmodium falciparum* is known to cause the majority of severe clinical disease. This parasite predominant in most of Indonesian islands over the second largest species *Plasmodium vivax.* The prevalence of *P. falciparum* compared to *P. vivax* in Sumatra is 3.5% vs. 2.9%, in Java/Bali: 3.2% vs. 2.6%, in Kalimantan: 5.4% vs. 3.4%, in Sulawesi: 4.2% vs. 2.7%, in Papua: 10.3% vs. 4.8% and in Maluku: 4.4% vs. 10.9% [2]. Significant attempts have been made to reduce morbidity by early diagnosis and prompt treatment, including the use of effective artemisinin combination therapy (ACT) since 2004, and careful case management to prevent onward transmission. Nonetheless, significant natural variation in *P. falciparum* transmission occurs in Indonesia along with variations in programmatic effectiveness.

Purworejo represents one of the hypo-endemic areas on Java island which successfully reduced *P. falciparum* prevalence to lower levels. Malaria control programmes are continuously updated in this district, which has reduced malaria incidence to less than one case/1,000 population/year, and which is defined as a low case incidence (LCI) area [3]. This is supported by data from the District Health Office which showed that the annual parasite incidence in Purworejo decreased from 1.66 cases per 1,000 population per year in 2004, to 0.42, 0.55, 0.57 and 0.61 cases per 1,000 population per year in 2005, 2006, 2007 and 2008, respectively. The other site, Lampung Selatan, represents a meso-endemic area [4]. Data from the District Health Office from 2004 to 2008 (recorded at health centre clinics and hospitals) show that the district of Lampung Selatan is a medium case incidence (MCI) area with between one to five malaria cases per 1,000 population per year.

**Figure 1:**
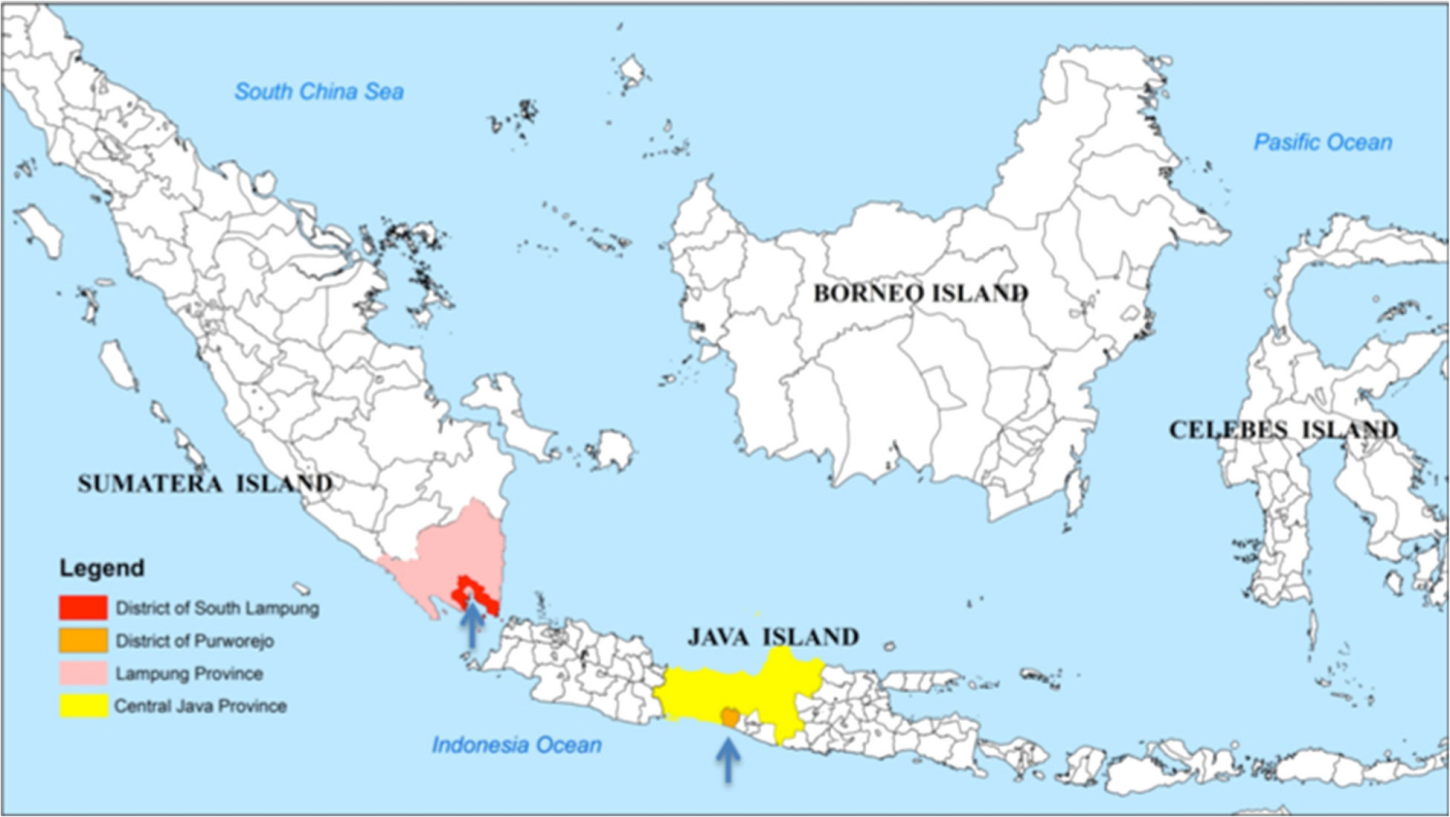
Study site: District of Purworejo (hypoendemic) and South Lampung (mesoendemic).

**Figure 2:**
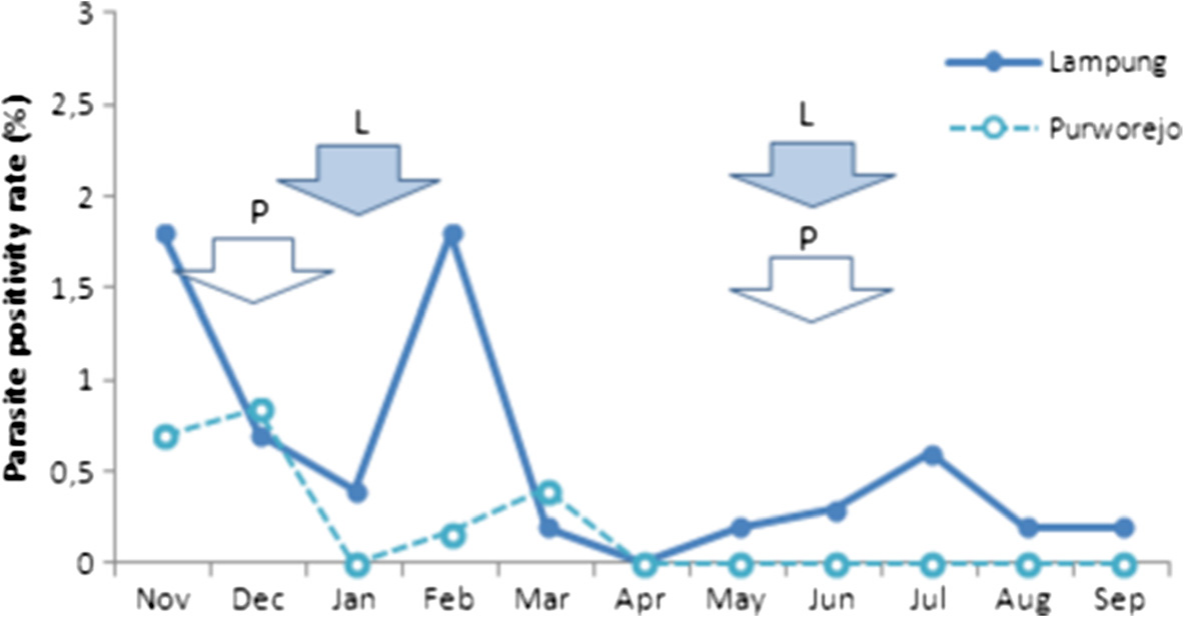
**The monthly slide positivity rate (SPR) from November 2008 to September 2009 in Lampung and Purworejo.** Arrows show the time of bloodspot collection during high (left) and low (right) season.

Transmission of malaria in Purworejo and in Lampung (Figure 1), as in most parts of Indonesia, occurs seasonally. Microscopic examination of blood smears collected during monthly cross-sectional surveys from October 2008 to September 2009 (Figure 2) indicates that the peak slide positivity rate (SPR) in Purworejo is between November 2008 and January 2009, while during the other months SPR is either very low or zero. In Lampung, the peak of SPR occurs between December 2008 and February 2009, and in other months SPR is considerably lower, but never zero. Therefore, estimating the level of malaria transmission based on microscopy in a low endemic region is far from accurate.

Naturally acquired antibody responses to malaria have been shown to develop after exposure [5,6]. As antibody responses may persist much longer than the parasite infection itself [7,8], they represent the footprint of an infection and have been used to estimate the levels of malaria exposure in some regions [9-11]. Methods for evaluating levels of malaria transmission using serological parameters have also been established in Indonesia, using recombinant protein of *P. falciparum* merozoite surface protein 1 (PfMSP1-19) and apical membrane antigen 1 (PfAMA1). Specific IgG antibody responses to these antigens have been demonstrated in a variety of malaria endemic areas and shown to have a strong correlation with estimates of entomological inoculation rate (EIR) [11,12].

In this study, antibody responses against these two merozoite antigens (PfMSP1-19 and PfAMA1) were used to assess differences between the two study areas as well as seasonal changes in exposure. Samples were collected in cross-sectional surveys from individuals of all ages during high and low season and both prevalence and magnitude of the responses were compared.

## Methods

### Study site and sample collection

The study was conducted in the hypo-endemic district of Purworejo, Province of Central Java, and in the meso-endemic district of South Lampung and Pesawaran, Province of Lampung (Figure 1). Purworejo district is located in the southern part of Java island with an Indian Ocean coastal area in the south, the Menoreh hilly areas in the eastern part, some foothills of the Dieng plateau at the north tier and a broad low plain of rice cultivation dominating the western part. Three subdistricts or “kecamatan” (Kaligesing, Bener and Loano), which in three previous years had a parasite incidence below one/1,000 population per/year were selected. As recorded in the District Statistic and Demographic Office, the total population of these three subdistricts is 102,562 people. The district of South Lampung is situated in the southern part of Sumatra island with coastal and a lowland areas dominating the southern and western parts, the northern and eastern parts represent a higher landscape, one part with cacao and coffee plantations and another part with hilly forested areas. Two subdistricts (Padang Cermin and Rajabasa), which in previous years had parasite incidences between one to five/1,000 population/year were selected with a total population of 82,000 people.

To evaluate differences in malaria transmission during high and low season blood samples were collected by house to house survey conducted during the peak of high season (November 2008 to January 2009) in Purworejo, (December 2008 to February 2009) in Lampung and during peak of low season (May to August 2009) both in Purworejo and in Lampung (determined base on monthly parasite rate data). Cluster random sampling technique was applied to select 165 household from each sites and season with assumption each household has 3 individual to cover a minimum sample size of 484. The number of samples was calculated using the formula of Daniel, 1999 [13] with confidence interval of 95%, precision 0.05, P value of 30% and design effect of 1.5. Blood spots were collected on Whatman 3 M paper (Whatman, UK) as described in [14] and were stored at −20°C until analysis. Individuals’ signed consent was obtained from all members of a selected household who agreed to participate in the study. For children under five years of age, consent was obtained from parents or guardians. Age-related ELISA tests were conducted upon blood spot samples collected: during the high season in Purworejo 527 samples in Lampung 490 samples, and during the low season in Purworejo: 488 samples, in Lampung 517 samples. All samples covered six age groups: one to four years, five to nine years, ten to 15 years, 16–25 years, 26–40 years, and >40 years. To evaluate parasite prevalence, thick blood smears collected during monthly malaria survey conducted from November 2008 to September 2009 in Lampung and in Purworejo were stained with 5% Giemsa and blood volume corresponding to at least 200 leucocytes was examined by a trained microscopist. Parasite density was calculated assuming 8000 leukocytes/ml.

### ELISA

The recombinant proteins *P. falciparum* MSP1-19 (Wellcome genotype*)* and *P. falciparum* AMA-1 (3D7) were used as antigens in indirect ELISA as described in [14]. Briefly, antigens were coated on plates at the concentration of 0.5 mg/mL in coating buffer and incubated at 4°C overnight. The plates were washed in PBST, and blocked with 1% (w/v) skimmed milk solution for three hours. After washing, samples were added in duplicate at a final dilution of 1:1,000 to each plate together with a pool of hyper immune serum and the plates were incubated overnight at 4°C. The plates were washed and 50 μl of HRP*-*conjugated rabbit anti human IgG (DAKO, #P0214) were added into each well and incubated for three hours. After a further series of washes, substrate solution (OPD, Sigma #P8287, in PBS) was added and the reaction was allowed to develop for 15–20 min before addition of stopping solution (2 M H2SO4). The optical density was read using ELISA reader at 450 nm.

### Statistical analysis

Raw optical density (OD) measurements were averaged and normalized against the positive control samples on each plate. Negative control sera were from 40 Javanese who reported no travel to malaria endemic areas. Visual assessment of results showed that log-transformed titer values of control group were approximately normal. The mean OD plus three standar deviation (SD) was used as the cut-off value for seropositivity [14]. A separate cut-off value was generated for each antigen. The difference between the seroprevalence of MSP-19 and AMA-1 antibodies during high transmission season and low transmission season in Purworejo and Lampung was analysed using Mantel-Haenszel and Chi-squared statistical tests. Differences in the magnitude of antibody responses were analysed using the Kruskal-Wallis or Mann–Whitney tests and shown as reverse cumulative distribution plots. Seroconversion rates (SCR) were estimated by fitting a simple reversible catalytic model [9] to seroprevalence data for each antibody in both sites in the high and low transmission season. Models with two (SCR) were fitted where appropriate. Reverse cumulative distribution plots [15] were used to examine the difference between the magnitude of antibody responses and both site and season.

## Results

### Characteristics of the study population

In Purworejo a total of 527 and 488 people were recruited during high and low season respectively, with a proportion of 49.1% male and 50.9% female, covering all ages from one to 78 years old. Interviews conducted during the survey indicated that 52.4% work as farmers or fishermen, and 9.3% as businessmen in the private sector; 43.5% of households had a bednet but only 14.8% of people used the bednet to sleep. In Lampung 490 and 517 people were recruited during high and low season, respectively. These were 49.3% male and 50.7% female, 22.7% worked as farmers or fishermen, and 5.7% as businesmen in the private sector; bednet ownership was at 93.8% and bednet usage at 57.7%.

### Antibody prevalence in Lampung and Purworejo during low and high transmission season

Differences in the levels of malaria exposure between meso-endemic Lampung and hypo-endemic Purworejo are reflected in the percentage of individuals who are seropositive for a particular antigen in each setting. Seroprevalence to anti-PfMSP1-19 or anti-PfAMA1 alone or combined is shown in Table 1. Seroprevalence was higher for both antigens in Lampung compared to Purworejo and this was observed in both the low and high seasons. Within sites, seroprevalences were higher in the high season compared to the low transmission season for both antigens in hypo-endemic Purworejo, but only for PfMSP1-19 in meso-endemic Lampung.

**Table 1:** Antibody prevalence to merozoite antigens by site and transmission season

**Site**	**Lampung**		**Purowejo**	
**Transmission season (N)**	**Low (517)**	**High (490)**	**Low (488)**	**High (527)**
**PfMSP1-19**	**33.7**	43.7 **	**10.0**	18.3**
**PfAMA1**	**37.5**	**41.0**	**14.5**	19.4*
**Either antigen**	**51.3**	**53.9**	**25.0**	37.5**

**p < 0.001, *p < 0.05 – designate chi-squared test between seasons for *each site.*

### Prevalence and magnitude of antibody responses to merozoite antigens by age group

**Figure 3:**
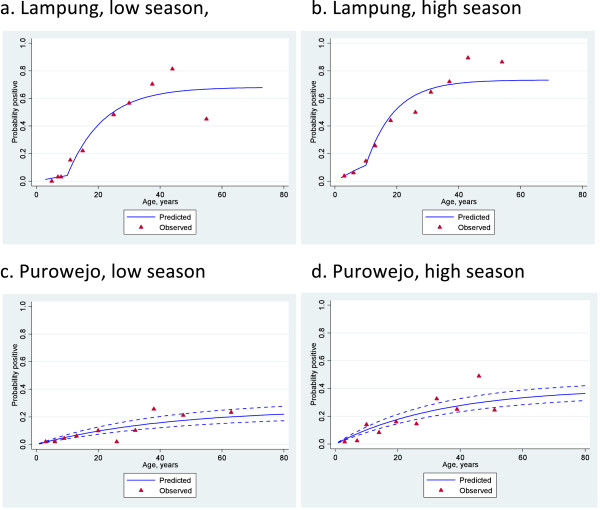
**Age seroprevalence plots with fitted seroconversion line for PfMSP1-19 in Lampung (a & b) and in Purowejo (c & d) during low and high season.** Red triangles indicate observed sero prevalence and solid blue lines show model-predicted seroprevalence. Broken blue lines are 95% confidence intervals.

The age-related differences in seroprevalence and MOD for both antibodies during low and high transmission season in meso-endemic Lampung are presented in Table 2. As expected, seroprevalence and MOD increase with age group in both sites and are higher in the high transmission season compared to the low transmission season. The difference in MOD is most apparent in the reverse cumulative distribution plots (Figure 3). The lowest seroprevalence and MOD are in antibody responses to PfMSP1-19 in children aged one to four years in Purworejo (1.5% and 0.066, respectively) (Table 3). Conversely, the highest seroprevalence and MOD are seen in individuals aged 40 and above in Lampung in the high transmission season (91.3% and 0.460, respectively) (Table 2).

**Table 2:** Prevalence and magnitude of antibody response to merozoite antigens by age group in Lampung

**Season**	**Antigen**	**Age group (N)**	**1-4**	**5-9**	**10-15**	**16-25**	**26-40**	**>40**
Low	PfMSP1-19	Prevalence	0 (62)	1.3(96)	15.5(84)	43.1(58)	61.8(123)	61.7(94)
		Mean OD	0.080	0.081	0.11	0.182	0.263	0.329
High	PfMSP1-19	Prevalence	6.9(72)	8.4(71)	22.1(68)	46.1(65)	63.4(134)	91.2(80)
		Mean OD	0.083	0.096	0.125	0.164	0.247	0.460
Low	PfAMA1	Prevalence	3.2(62)	3.2(96)	22.6(84)	43.1(58)	66.7(123)	67.0(94)
		Mean OD	0.091	0.078	0.111	0.161	0.272	0.324
High	PfAMA1	Prevalence	6.9(72)	14.1(71)	20.6(68)	47.7(65)	58.2(134)	78.7(80)
		Mean OD	0.072	0.100	0.111	0.168	0.247	0.429
Low	Combined	Prevalence	5.0(62)	16.7(96)	38.1(84)	58.6(58)	78.5(123)	80.5(94)
High		Prevalence	16.8(72)	25.3(71)	44.1(68)	60.0(65)	66.4(134)	95.0(80)

**Table 3:** Prevalence and magnitude of antibody response to merozoite antigens by age group in Purworejo

**Season**	**Antigen**	**Age group (N)**	**1-4**	**5-9**	**10-15**	**16-25**	**26-40**	**>40**
Low	PfMSP1-19	Prevalence	1.6(62)	1.3(77)	7.6(66)	8.5(71)	12.7(115)	22.7(97)
		Mean OD	0.067	0.071	0.085	0.093	0.113	0.142
High	PfMSP1-19	Prevalence	5.6(71)	5.4(74)	8.1(74)	18.4(76)	23.7(118)	35.1(114)
		Mean OD	0.081	0.091	0.104	0.133	0.154	0.192
Low	PfAMA1	Prevalence	3.2(62)	5.4(77)	12.1(66)	12.7(71)	14.8(115)	32.0(97)
		Mean OD	0.059	0.064	0.085	0.093	0.101	0.148
High	PfAMA1	Prevalence	5.6(71)	6.8(74)	6.8(74)	17.1(76)	23.7(118)	41.2(114)
		Mean OD	0.079	0.076	0.084	0.138	0.146	0.191
Low	Combined	Prevalence	4.8(62)	9.1(77)	16.7(66)	21.1(71)	30.4(115)	52.6(97)
High		Prevalence	11.2(71)	18.9(74)	27.0(74)	42.1(76)	50.0(118)	57.0(114)

### Parasite rates in Lampung and Purworejo

The *P. falciparum* SPR in Lampung and in Purworejo obtained during monthly malaria surveys from November 2008 to September 2009 show the seasonal fluctuation of transmission (Figure 2). Lampung has a higher *P. falciparum* SPR with a peak at 1.8% in November and February; SPR is lower from April onward but never zero. In Purworejo, a peak SPR of only 0.84% is reached in December, and from April onwards no parasites were found in the surveys. Blue arrows indicate the time points during which the majority of inhabitants in the study area were screened by microscopy for malaria and treated as appropriate.

### Analysis of seroconversion rates by site and season

**Figure 4:**
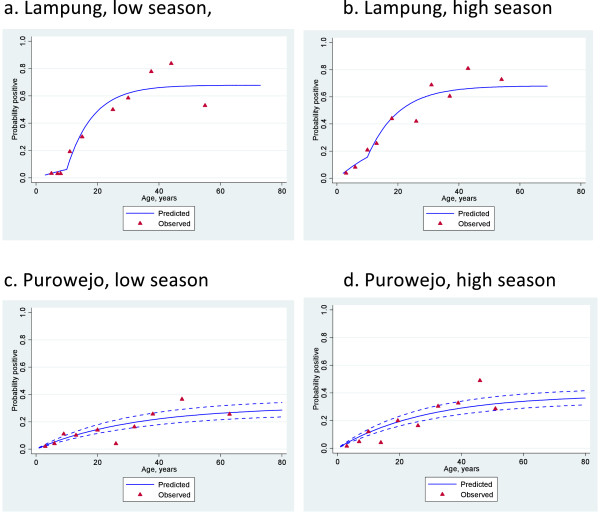
**Age seroprevalence plots with fitted seroconversion line for PfAMA1 in Lampung (a & b) and in Purowejo (c & d) during low and high season.** Red triangles indicate observed sero prevalence and solid blue lines show model-predicted seroprevalence. Broken blue lines are 95% confidence intervals.

**Figure 5:**
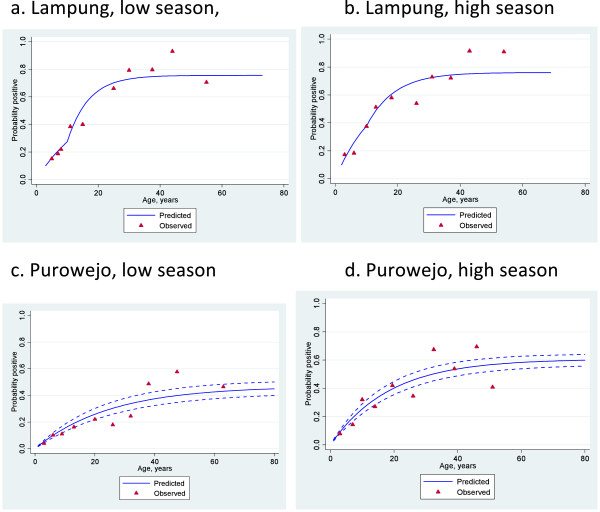
**Age seroprevalence plots with fitted seroconversion line for combined (PfMSP1-19 and PfAMA1) antibody response in Lampung (a & b) and in Purowejo (c & d) during low and high season.** Red triangles indicate observed sero prevalence and solid blue lines show model-predicted seroprevalence. Broken blue lines are 95% confidence intervals.

**Figure 6:**
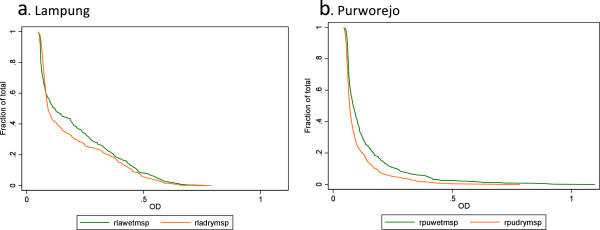
**Reverse cumulative distribution plots to compare antibody responses to *P. falciparum *** **MSP1-19 between seasons in Lampung (a) and Purworejo (b).**

The relationship between age and seroprevalence was examined using catalytic conversion models. The age seroprevalence plots are shown in Figures 4, 5 and 6 and the corresponding seroconversion rates are shown in Table 4. As was observed for the overall seroprevalence, the SCR for PfMSP1-19 was significantly different between the low and high transmission seasons in Purworejo as evidenced by the non-overlapping confidence intervals.Using the conversion of Corran *et al.*[11], these SCR values equate to entomological inoculation rates (EIR) of approximately 0.05 infectious bites per person per year (ib/p/yr) in the low season to 0.2 ib/p/yr in the high season, i.e., a four-fold increase. A similar difference is seen with the SCR to PfAMA-1, but this is not statistically significant. However, SCRs, based on the combined antibody prevalence, also show a significant difference between the high and low season although there are no EIR equivalents for this measure.

**Table 4:** **Seroconversion rates for *Plasmodium falciparum *** **merozoite antigens using age specific sero-prevalence**

**Site**	**Lampung**^**#**^		**Purowejo**	
**Transmission season (N)**	**Low Lambda (95% CI)**	**High (490)**	**Low (488)**	**High (531)**
^a^PfMSP1-19				
Current	0.004 (0.001-0.01)	0.013 (0.007-0.02)	0.006 (0.004-0.008)	0.011 (0.009-0.014)
Previous	0.067 (0.05-0.087)	0.091 (0.07-0.12)		
^b^PfAMA1				
Current	0.007 (0.003-0.014)	0.019 (0.011-0.031)	0.009 (0.007-0.012)	0.013 (0.01-0.016)
Previous	0.096 (0.072-0.12)	0.078 (0.056-0.11)		
^b^Combined				
Current	0.035 (0.026-0.049)	0.053 (0.039-0.073)	0.019 (0.015-0.022)	0.032 (0.03-0.038)
Previous	0.124 (0.084-0.18)	0.091 (0.058-0.14)		

^#^for Lampung a model with two separate forces of infection (with a change point 10 years before present) fitted the data better than a model with a single force of infection.

^a^for PfMSP1-19 the rate of reversion in the seroconversion models was restricted to 0.017.

^b^for PfAMA1 and the combined antibody response the rate of reversion in the seroconversion models was restricted to 0.026.

Interestingly, the SCR models for Lampung all demonstrated a significantly better fit when two SCRs were calculated with a step in the seroconversion curve at ten years. This was observed for both antigens separately and when combined and in both the high and low seasons (Figures 3, 4 and 5). EIR equivalents suggest an approximate five-fold difference in transmission in individuals below and above the age of ten, with those below ten years old experiencing <0.1 ib/p/yr in the low season and those above, 2ib/p/yr. These values are doubled in the high transmission season.

**Figure 7:**
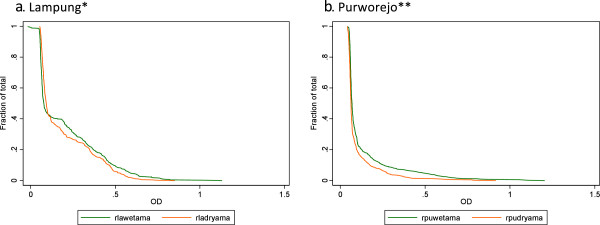
**Reverse cumulative distribution plots to compare antibody responses to *P. falciparum *to AMA-1 between seasons in Lampung and Purworejo. a**. Lampung*) Wilcoxon rank sum test shows significant difference in antibody levels between season (P < 0.001). **b**. Purworejo**) Wilcoxon rank sum test shows significant difference in antibody levels between season (P < 0.0001).

The seasonal difference was also apparent when comparing the magnitude of individual antibody responses in meso-endemic Lampung and hypo-endemic Purworejo. The range of optical densities is shown in reverse cumulative distribution plots in Figures 6 and 7 for MSP1-19 and AMA1, respectively. The relevant Wilcoxon rank sum test in (Figure 7) showed significantly higher OD in the high transmission season for both antigens in both sites.

## Discussion

In recent years, serology to detect anti-malarial antibody responses has proven useful to evaluate the level of malaria transmission intensity, especially in low endemic areas. This is in part due to the ready availability of standardized antigens such as recombinant *P. falciparum* MSP-1_19_ (Wellcome genotype) and AMA-1 (3D7) antigens [9-11,16] as opposed to using crude parasite extracts. In this current study, these methods were used to examine the stability of antibody responses of people living in areas of hypo- and meso-endemic malaria in Indonesia. The data show that antibody responses as either prevalence, SCR or magnitude of antibody response are able to differentiate between areas of different transmission intensity as has been previously described [10,12,16]. The analysis also showed that within each site these three outputs were able to differentiate between the low and high transmission seasons. These seasonal fluctuations are most prominent in low transmission Purworejo and clearer with antibody responses to PfMSP1-19.

This observation of the antibody response reflecting seasonal patterns of malaria has not been consistently reported in the literature, which is particularly true for seroprevalence. Antibody responses are likely to persist for markedly longer than infections and data suggest that responses can last for several years if not decades reviewed in [17]. This would suggest seroprevalence would be relatively insensitive to short-term fluctuations in transmission. However, serological surveys from Cambodia observed population level increases in SCR by approximately two-fold between low and high season [18]. One explanation is that in low transmission settings antibody responses are less well-established, such that fluctuations of antibody density around the nominal threshold for seropositivity [19] are more likely to occur. This would also be influenced by the immunogenicity of the antigen of choice. In this study, higher overall responses were observed to PfAMA-1 with no statistical differences in SCR between seasons in either site. Conversely, SCR were significantly different between seasons for PfMSP1-19. PfMSP-1 is a smaller molecule than PfAMA-1 and likely has fewer epitopes for antibody binding such that fewer anti-PfMSP1-19 responses are generated [11]. Similar observations have also been made in antibody responses to PfMSP1-19 in children in The Gambia [20,21]. This hypothesis is further supported to some degree by the seasonal differences in the reverse cumulative distribution plots (RCDP) [11,21] of the optical densities of the antibody responses. Antibody responses are clearly different between the high and low season surveys with more profound changes in PfMSP1-19 responses compared with PfAMA1 though both being significant. Validation of such observations requires further surveys in areas of low seasonal malaria transmission.

Serological outputs clearly defined Lampung as a higher transmission setting than Purworejo with overall seroprevalence between two- and 1.5-fold higher in Lampung depending on the season. In Lampung, a catalytic conversion model in which two forces of infection were used gave a better fit than a model with a single force of infection. This may either point to a pronounced change in transmission intensity ten years ago (e.g. due to an intervention), or alternatively, to a change in the force of infection at ten years of age (e.g. due to a change in behaviour). A reversible catalytic model fitted to data from a single cross-sectional survey cannot distinguish between the two hypotheses, but epidemiological evidence favours an age related change in exposure. The most likely explanation seems that transmission is associated with some behavioural risk such as frequenting coastal areas for fishing where local vectors such as *Anopheles sundaicus* predominate in the south part of Lampung and/or for rubber tapping to forested areas where *Anopheles kochi* and *Anopheles punctulatus* are present in the northern part of Lampung. This behaviour change has been documented in other areas in Southeast Asia, such as Cambodia [18], where both social science and biological indicators implicate a significant increase in exposure with proximity to and/or travel to areas where local vectors persist. Temporal changes in socio-economic status of the population in Lampung may also be a determinant factor that contribute to the changes in malaria exposure as has been reported in the population in Central Vietnam [22], where the wealthier people are less exposed. Another factor that might also contribute to those changes is that South Lampung is one of the malaria-endemic areas in Indonesia that is the site of ongoing malaria research projects. Based on the District Health Office records (pers comm), case finding followed by standard treatment has been done almost every year for the last eight years although no formal evaluation of this approach has been carried out. The impact of mass screening treatment on malaria burden and transmission has been clearly documented in studies in west Kenya [23] and other part of Africa [24], although combination with other measures is needed for longer-term effect [25].

Together these data suggest that serological analysis could be an important additional tool for malaria monitoring and surveillance in Indonesia and elsewhere. The observations of differential responsiveness of specific merozoite antigens to changes in exposure further strengthens the notion that differences in antigen immunogenicity could be tailored to specific use scenarios reflecting long, medium and short term exposure and a serological marker(s) of disease incidence [11]. Techniques such as microarray [26] and bead based flow assays [27] will go a long way to identifying and validating potential antigens for these applications. At the moment these techniques may have a limited availability to control programmes and the ELISA remains more amenable. The measurement of the differential kinetics of antibody responses may also prove more useful as the data show differences both in seroprevalence and antibody levels as seasons. Both historical [28] and more recent studies [29] have shown that, not surprisingly, antibody levels drop before seroprevalence is affected. The development of a standardized assay and analysis will facilitate in determining just how useful antibody level measurements are.

## Conclusion

Antibody responses to recombinant *P. falciparum* merozoite surface antigens demonstrate the utility in describing differences in endemicity and seasonality in these hypo/meso-endemic areas of Indonesia. This demonstrates the wider potential for the use of serological responses in monitoring changes in transmission as countries switch from control to elimination strategies.

## Competing interests

The authors declare that they have no competing interests.

## Authors’ contributions

SSp designed the study, coordinated the field work in Purworejo and coordinated laboratory works and wrote the manuscript; MTB assisted in analysing data, discussed the results and assisted in writing the manuscript; MAW coordinated blood sample collection and microscopic blood slide examination; IS and AAK coordinated and conducted blood sample collection and microscopic examination in Lampung; DN and RR conducted sampling procedure, coordinated sample collection, performed the ELISA assays, inputed the data and conducted statistical analysis; NFL assisted in importing the antigens, designing and conducting of the study; WAH assisted with study design and revised the manuscript; SSu coordinating GPS and statistical analysis, JC assisted in laboratory analyses, processed the raw data and performed initial statistical analyses; CJD assisted in designing the study, conducting data analysis and writing manuscript, providing and standardising the antigens. All authors have read and approved the final manuscript.
